# SARS-CoV-2 Variants by Whole-Genome Sequencing in a University Hospital in Bangkok: First to Third COVID-19 Waves

**DOI:** 10.3390/pathogens12040626

**Published:** 2023-04-21

**Authors:** Chayanee Setthapramote, Thanwa Wongsuk, Chuphong Thongnak, Uraporn Phumisantiphong, Tonsan Hansirisathit, Maytawan Thanunchai

**Affiliations:** 1 Department of Clinical Pathology, Faculty of Medicine Vajira Hospital, Navamindradhiraj University, Bangkok 10300, Thailand; 2 Department of Central Laboratory and Blood Bank, Faculty of Medicine Vajira Hospital, Navamindradhiraj University, Bangkok 10300, Thailand; 3 Division of Clinical Microbiology, Department of Medical Technology, Faculty of Associated Medical Sciences, Chiang Mai University, Chiang Mai 50200, Thailand

**Keywords:** SARS-CoV-2, COVID-19, whole-genome sequencing, genetic diversity, mutation, disease severity

## Abstract

Background: Multiple severe acute respiratory syndrome coronavirus 2 (SARS-CoV-2) variants emerged globally during the recent coronavirus disease (COVID-19) pandemic. From April 2020 to April 2021, Thailand experienced three COVID-19 waves, and each wave was driven by different variants. Therefore, we aimed to analyze the genetic diversity of circulating SARS-CoV-2 using whole-genome sequencing analysis. Methods: A total of 33 SARS-CoV-2 positive samples from three consecutive COVID-19 waves were collected and sequenced by whole-genome sequencing, of which, 8, 10, and 15 samples were derived from the first, second, and third waves, respectively. The genetic diversity of variants in each wave and the correlation between mutations and disease severity were explored. Results: During the first wave, A.6, B, B.1, and B.1.375 were found to be predominant. The occurrence of mutations in these lineages was associated with low asymptomatic and mild symptoms, providing no transmission advantage and resulting in extinction after a few months of circulation. B.1.36.16, the predominant lineage of the second wave, caused more symptomatic COVID-19 cases and contained a small number of key mutations. This variant was replaced by the VOC alpha variant, which later became dominant in the third wave. We found that B.1.1.7 lineage-specific mutations were crucial for increasing transmissibility and infectivity, but not likely associated with disease severity. There were six additional mutations found only in severe COVID-19 patients, which might have altered the virus phenotype with an inclination toward more highly pathogenic SARS-CoV-2. Conclusion: The findings of this study highlighted the importance of whole-genome analysis in tracking newly emerging variants, exploring the genetic determinants essential for transmissibility, infectivity, and pathogenicity, and helping better understand the evolutionary process in the adaptation of viruses in humans.

## 1. Introduction

Three years have passed since the world faced the major challenges caused by the coronavirus disease (COVID-19) crisis; when the crisis will end remains unknown. The disease is caused by severe acute respiratory syndrome coronavirus 2 (SARS-CoV-2), which first appeared in Wuhan, China, in late December 2019. Since then, it has infected more than 700 million people and caused over 6 million deaths globally [1]. The more the virus circulates, the greater the chance for mutations to occur; as a process, viral evolution randomly generates a large number of variants with either more or fewer pathogenic characteristics [2,3]. Genomic diversity based on whole-genome sequencing (WGS) data can precisely identify evolving variants and their hotspot mutations, which is beneficial for tracking the spread of the virus, predicting disease outcomes, and guiding vaccine and therapeutic development [4].

According to the current situation, the omicron variant (B.1.1.529) and its sub-lineages are designated as variants of concern (VOC) and variants of interest (VOI). Only the Omicron sub-lineage BA.2 listed in VOC harbors mutations that have a potential impact on increased transmissibility. All VOC and VOI omicron sub-lineages exhibited reduced severity but increased immune evasiveness. Therefore, the effectiveness of vaccines to protect against omicron was reduced [5,6]. Previous VOCs that were once a global threat, such as alpha (B.1.1.7), beta (B.1.351), gamma (P.1), and delta (B.1.672), are listed as de-escalated variants due to no longer circulating and having little impact on the current epidemiological situation. The circulating VOC omicron variant shares some mutations with the alpha (S:N501Y, P681H) and delta (S:T478K, T478K, E484A) variants, which have been proven to be evolutionary selective benefits for the virus [7,8].

In Thailand, we encountered five COVID-19 surges over the past three years, resulting in a cumulative total of 4 million confirmed cases and 33,882 deaths [9]. Several variants and lineages were introduced to regions in various ways, such as human travel, the migrant movement of workers, and crowded entertainment venues. The first wave of COVID-19 initially spread at boxing stadiums and nightlife venues in March 2020, with 3042 cases and 57 deaths being reported [10]. A.6 and B.1 were the dominant lineages. Most of the patients had asymptomatic and mild upper respiratory symptoms, such as fever, cough, and sore throat. The number of confirmed cases in the first wave subsided in May 2020 due to the successful implementation of public health and social measures as well as a full lockdown [11,12]. Six months later, a large number of illegal migrant workers crossed the borders into Thailand for work and carried the B.1.36.16 lineage with them. Due to having asymptomatic or mild symptoms, migrant workers might have inadvertently spread the virus, thus triggering the second wave, which caused over 20,000 confirmed cases in 2.5 months [10,13]. The successful containment of the pandemic throughout the year was interrupted by the third wave outbreak in April 2021, when alpha-B.1.1.7 was the main variant. The outbreak started in pubs, bars, and restaurants, resulting in 127,000 cases throughout Thailand, which was six times greater than that in the second wave [10]. From July 2021 to December 2021, the fourth wave was encountered, which was driven by the highly contagious delta-B.1.672 variant. This surge was the worst outbreak in terms of having the highest infection rates and death toll, resulting in the collapse of the public health system [14]. At the time of preparing the manuscript, we were living with omicron sub-lineages, for which the cumulative case numbers were greater than the previous outbreak, though the symptoms caused by these variants were milder [15]. Over 70% of Thailand’s population was fully vaccinated [9]. People with confirmed or suspected COVID-19 could be isolated at home, and all preventive measures were implemented continuously.

Before the mass COVID-19 vaccination roll-out in Thailand, SARS-CoV-2 variants from the first, second, and third waves spread extensively among populations who were not immune to the disease. The mutations that emerged during this period were likely to develop independently of immune selective pressures. Therefore, this study aimed to explore the genotypes of SARS-CoV-2 lineages that circulated from the first to the third waves. We performed WGS using clinical samples collected from Vajira Hospital, Bangkok, Thailand between April 2020 and April 2021. Viral mutations associated with disease severity were characterized.

## 2. Materials and Methods

### 2.1. Ethical Approval

This study was approved by The Ethical Review Board of the Faculty of Medicine, Vajira Hospital, Navamindradhiraj University (approval ID COA 049/2563). The informed consent was waived due to all samples being anonymous.

### 2.2. Sample Selection and Viral RNA Extraction

Forty nasopharyngeal swab (NPS) specimens of COVID-19-suspected patients routinely sent for diagnosis at the Molecular Laboratory Unit, Division of Central Laboratory and Blood Bank, Faculty of Medicine, Vajira Hospital, Navamindradhiraj University from April 2020 to April 2021 were selected for RNA extraction and WGS. Viral RNA of COVID-19 cases confirmed by a routine method were extracted using QIAamp Viral RNA Mini kit (QIAGEN, Qiagen, Germantown, MD, USA) following the manufacturer’s instructions.

### 2.3. Collection of Demographic and Clinical Data

The demographic and clinical data, including sex, age, clinical features, inpatient/outpatient status, and comorbidities, were retrieved from medical records. The disease severity was classified as asymptomatic, mild, moderate, severe, and critical illnesses according to World Health Organization; asymptomatic (laboratory-confirmed patients without COVID-19 symptoms), mild (fever and upper respiratory symptoms with no signs of pneumonia), moderate (fever and respiratory symptoms with evidence of lower respiratory disease), severe (oxygen saturation < 94%, ratio of arterial partial pressure of oxygen to fraction of inspired oxygen < 300 mm Hg, respiratory rate > 30 breaths/min, or lung infiltration > 50%), and critical (respiratory failure, septic shock, and/or multiple organ failure) [16].

### 2.4. WGS and Phylogenetic Tree

#### 2.4.1. WGS of the First Wave

Nine samples from April 2020 were subjected to WGS using an Atoplex System, in accordance with the manufacturer’s protocol. In brief, 10 uL of RNA were reverse transcribed to cDNA. Then, the DNA library which contained a Dual barcode adaptor was performed by two-step multiplex PCR to amplify the RNA target region. The pooled DNA library was used to create circularized single strand DNA (ssCirDNA) by following the protocol of ATOPlex RNA Universal Library Preparation Kit (MGI Tech Co., Ltd., Shenzhen, China). DNA nanoball (DNB) was generated from ssCirDNA for subsequent sequencing on MGISEQ-2000RS sequencer by following the protocol of MGISEQ-2000RS High-throughput Sequencing Set (MGI Tech Co., Ltd., Shenzhen, China). The sequences were cleaned and retrieved by the bioinformatician at the Medical Genome Company, Bangkok, Thailand.

After obtaining sequencing read in FASTQ format, the bioinformatic analysis is done by the iGVD software following the iGenomeVirusDetector User Guide (Genome Wisdom (Beijing, China) Gene Technology Co., Ltd.).

#### 2.4.2. WGS of the Second and Third Waves

A total of 31 samples collected during December 2020 to April 2021 were analyzed using the Ion Proton system (Thermo Fisher Scientific, Waltham, MA, USA) following the manufacturer’s protocol. The Ion AmpliSeq SARS-CoV-2 research panel used in this study contains 247 amplicons in two pools targeting the SARS-CoV-2 genome with >99% coverage. The library preparation of amplified samples was performed on the Ion OneTouch 2 System and then sequenced on the Ion Proton system (Thermo Fisher Scientific, Waltham, MA, USA) at the Center of Medical Genomics, Ramathibodi Hospital, Mahidol University, Thailand.

For data analysis, the sequencing reads were processed with the Ion Proton software plug-ins coverageAnalysis, IRMAreport, AssemblerTrinity, variantCaller (Torrent Suite software v5.20, with germline low-stringency settings according to the TS5.20 user guide), and GenerateConsensus (Ion AmpliSeq SARS-CoV-2 Insight research assay). Coverage analysis was set to a minimum depth of 30 reads. The consensus sequence was obtained directly from the IRMAreport and GenerateConsensus as the fasta file format. The consensus sequences were assessed using NextClade (https://clades.nextstrain.org/, version 1.9.0, accessed on 1 October 2021) for sequence clade assignment, identification quantification of mutations, and sequence quality analyses.

#### 2.4.3. Phylogenetic Tree

To assess the evolutionary relationships among the studied sequences, we aligned the sequence using an online version of MAFFT version 7 (https://mafft.cbrc.jp/alignment/server/, accessed on 13 February 2022). Then, evolutionary history was inferred using the maximum likelihood method. The best models of evolution for the dataset were selected from the Bayesian Information Criterion (BIC) of MEGA 11: Molecular Evolutionary Genetics Analysis across computing platforms [17]. The model with the lowest BIC score was selected to construct a maximum likelihood phylogenetic tree. The percentage of trees in which the associated taxa clustered together was shown next to the branches. The initial tree for the heuristic search was automatically obtained by the application of the Neighbor-Join and BioNJ algorithms to a matrix of pairwise distances estimated using the maximum composite likelihood (MCL) approach. We then selected the topology associated with a superior log likelihood value. The tree was drawn to scale, with branch lengths representing the number of substitutions per site. A bootstrap analysis was conducted using 500 replicates, and bootstrap values  ≥50% are shown above branches.

### 2.5. SARS-CoV-2 Lineage Classification

Consensus FASTA files of SAR-CoV2 genomic sequences were uploaded to the Pangolin web service to assign the most likely SARS-CoV-2 lineage to our samples (Pango nomenclature) [18]. CoVsurver online server (CoVsurver—CoronaVirus Surveillance Server, 2021) was used for GISAID clade assignment.

## 3. Result

### 3.1. Distribution of Three Waves of COVID-19 Outbreaks in Thailand

**Figure 1:**
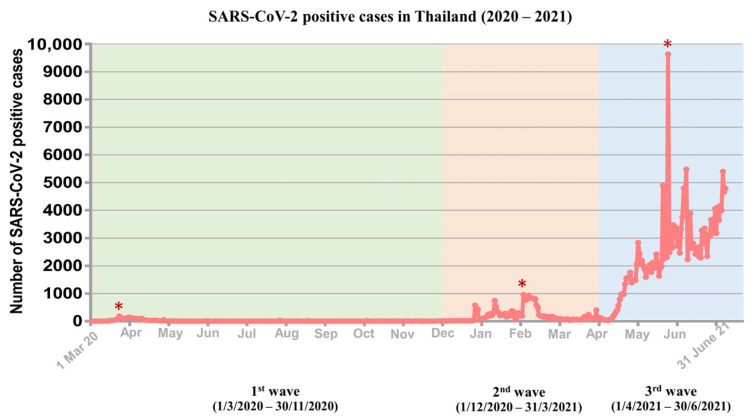
SARS-CoV-2 positive cases in Thailand. Graph based on a data source available at https://ourworldindata.org/coronavirus/country/thailand (accessed on 30 November 2021). Color of plotting area illustrates the sample collection time range for each wave during the COVID-19 pandemic. The asterisk indicates the highest number of new cases.

The COVID-19 situation in Thailand was divided into distinct waves driven by different dominant SARS-CoV-2 strains. In this context, the first (March 2020–November 2020), second (December 2020–March 2021), and third (April 2021–June 2021) waves were studied. The first wave of the SARS-CoV-2 outbreak started in early March 2020 and peaked between the 22 and 31 March 2020 (188 cases/day). The number of confirmed cases never exceeded 200 cases per day. For the second wave, a peak of transmission (959 cases/day) started on the 26 January 2021 until the 4 February 2021 with less than 1000 new COVID-19 cases per day. At the beginning of the third wave in early April 2021, the number of cases started to increase rapidly. A record of 9635 new cases, which was the highest daily number of cases since the pandemic began, was reported on 17 May 2021. The average number of new COVID-19 cases reported per day during this wave was nearly 3000 (Figure 1).

A total of 40 NPS swabs were collected from 40 confirmed COVID-19 patients who acquired the virus by local transmission and were subjected to WGS study. Given the poor genomic coverage (<95%), seven samples were excluded from the analysis. Thirty-three samples passed the Chi-square test performed by IQ-TREE multicore version 1.6.12 and were analyzed to represent the first, second, and third waves of the SARS-CoV-2 outbreak in the hospital (Table 1). The samples collected from travelers or residents returning home in the state quarantine were not included in the study. Viral lineages were assigned using Pangolin online software (https://cov-lineages.org/pangolin.html; accessed on 30 November 2021), respectively. Four viral Pangolin lineages (A.6, B, B.1, and B.1.375) were identified in eight samples collected during the first wave. All ten samples obtained during the second wave belonged to the B.1.36.16 lineage. This lineage disappeared quickly and was replaced by B.1.1.7 in approximately three months. Thus, all 15 samples collected in the third wave were B.1.1.7 and Q lineages (14 for B.1.1.7 and 1 for Q.3).

**Figure 2:**
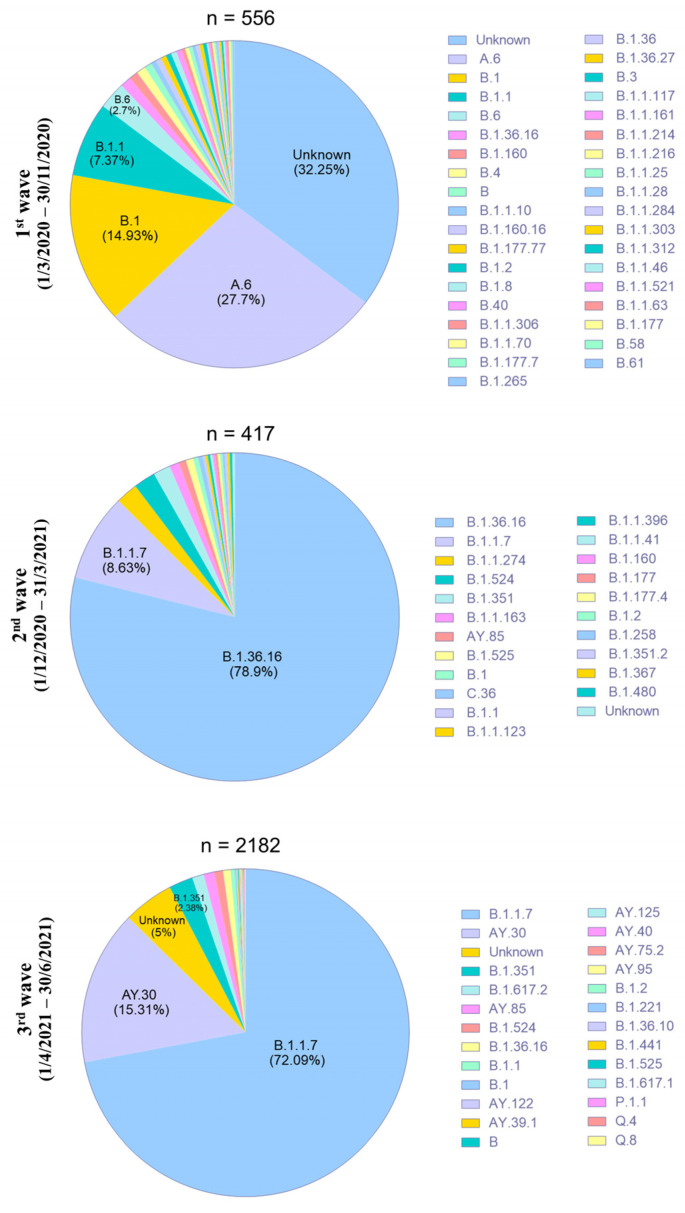
Distribution of SARS-CoV-2 Pangolin lineages in the first, second, and third COVID-19 waves in Thailand. Graph based on a data source available at https://cov-spectrum.org (accessed on 30 November 2021).

The shared SARS-CoV-2 data were retrieved by CoV spectrum, showing that 27.7% of A.6 in the first wave, 78.9% of B.1.36.16 in the second wave, and 72.1% of B.1.1.7 in the third wave were sequenced from clinical samples in Thailand and submitted to the database (Figure 2). Thus, A.6, B.1.36.16, and B.1.1.7 were the predominant lineages that circulated in the first, second, and third waves, respectively. Of note, B.1.36.16 was detected in very low frequency (1%) during the first wave, while a small number (8.63%) of B.1.1.7 was found in the second wave, demonstrating that these lineages had already emerged and circulated for a few months before becoming dominating variants of the second and third waves. The B.1.375 and Q.3 found in the samples for the current study had not been previously recorded in Thailand’s database.

### 3.2. Phylogenetic Tree

**Figure 3:**
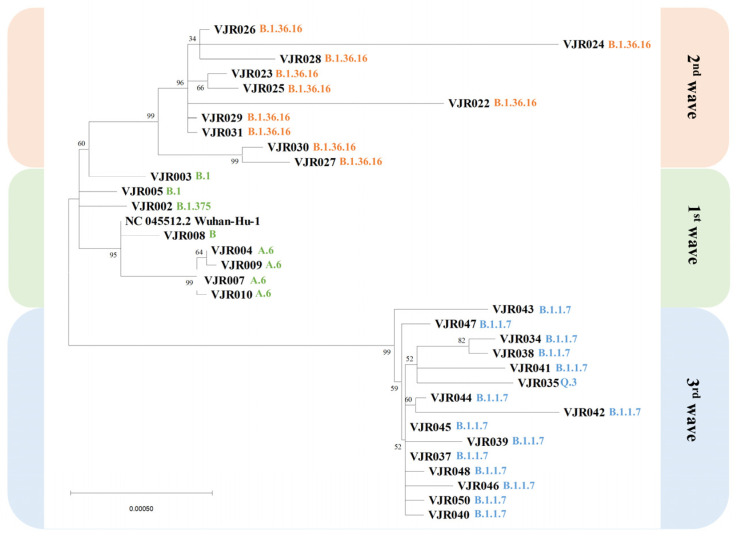
Phylogenetic tree of SAR-CoV-2 sequences. The percentage of trees in which the associated taxa clustered together is shown next to the branches. The initial tree for the heuristic search was obtained automatically by applying Neighbor-Join and BioNJ algorithms to a matrix of pairwise distances estimated using the MCL approach, and the topology with a superior log likelihood value was selected. The tree was drawn to scale, with branch lengths measured in the number of substitutions per site.

This study assessed the genetic relationship in the samples by building a phylogenetic tree in MEGA11 [17]. The T93:Tamura–Nei parameter model and the nonuniformity of evolutionary rates among sites can be modeled by using a discrete gamma distribution (+G), with five rate categories selected based on the best model of evolution analyses (BIC score: 87244.0255270594). Subsequently, a phylogenetic tree was constructed by maximum likelihood analysis based on the T93 + G model with 500 replications of bootstrap analysis. The tree with the highest log likelihood (−43190.83) was shown. A + G was used to model the evolutionary rate differences among sites [5 categories (+G, parameter = 0.0500)]. As a tree (Figure 3), 34 nucleotide sequences were involved. The codon positions included were 1st + 2nd + 3rd + Noncoding. A total of 30,266 positions were included in the final dataset.

The evolutionary analysis revealed the circulation of B, B.1, B.1.375, and A.6 lineages during the first wave of the selected samples at the hospital and demonstrated genetic relations to the Wuhan-Hu-1 isolate (NC_045512.2). Meanwhile, B.1.36.16 and alpha (B.1.1.7 and Q lineages) were detected during the second and third waves of infection at the hospital, respectively.

### 3.3. Characterization of SARS-CoV-2 Variants in Thailand

**Figure 4:**
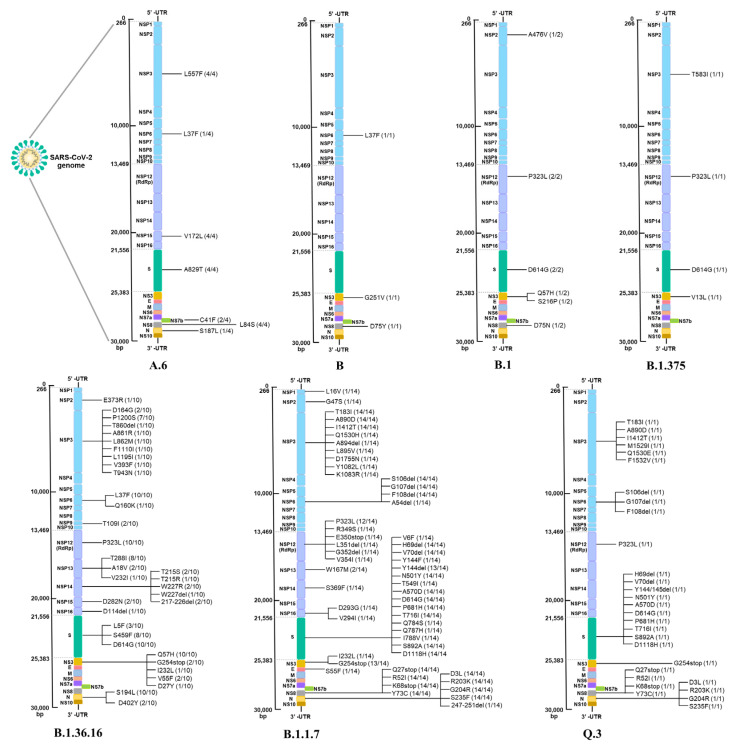
Changes in the aa of the protein in SARS-CoV-2 VOCs deciphered by WGS in this study. A.6, B, B.1, and B.1.375 variants were detected during the 1st wave of the pandemic. For the 2nd wave, all detected variants were B.1.36.16. In the 3rd wave, a large number of detected variants were mostly dominated by B.1.1.7, followed by the Q.3 variant. The number in the bracket represents the frequency of occurrence of an event (Number count/Total number of sequences classified in each lineage).

We further characterized the aa changes in each lineage. Figure 4 shows the mutations throughout the genome of each lineage in all three COVID-19 waves. Since A.6, B, B.1, and B.1.375 were the earliest clusters in Thailand, low levels of genomic diversity were expected in these lineages. In comparison to the reference strain Wuhan-Hu-1, there were less than 10 mutations in an entire genome. Focusing on the S protein, there was only one mutation (A829T) in the A.6 lineage, while D614G was initially detected in the B.1 and B.1375 lineages and remained present in all the lineages that circulated in Thailand. Similar to D614G, P323L in nonstructural protein (Nsp) 12 was first identified in the first wave (B.1 and B.1.375) and frequently detected throughout the period of study.

More mutations were observed in the second wave; B.1.36.16 carried 32 mutations, in which a high frequency was located on Nsp; there were nine mutations in Nsp3 and 15 mutations in Nsp14 (12 of these were deletions at positions 216–227). However, only three mutations (L5F, S459F, and D614G) were identified on the S protein. L37F in Nsp6 and Q57H in NS3, which were found in few samples from the first wave, later became predominant mutations of the second wave.

Notably, the B.1.1.7 lineage in the third wave harbored a large number of mutations. A total of 54 aa substitutions were identified throughout the genome, where the majority of mutations were located on the S protein. Mutation patterns on both S and other proteins, such as for example (i) S: del69-70HV, del144Y, N501Y, A570D, D614G, P681H, T761I, S982A, and D1118H; (ii) N: D3L, R203K, G204R, and S235F; (iii) Nsp3: T183I, A890D, and I1412T; (iv) Nsp6: del106-108SGF; (v) Nsp12: P323L; (vi) NS3: G254stop; and (vii) NS8: Q27stop, R52I, K68stop, and Y73C, were found with a high frequency and could define the specific lineage. Thus, these were recognized as lineage-defining mutations. The Q.3 lineage, which was sequenced from one sample during the third wave, contained lineage-defining mutations in all regions without any uncommon aa changes detected. 

The global occurrence of all mutations found in this study are shown in the Supplementary Data section. Similar to high global frequency, S: D614G and Nsp12: P323L were detected in 28/33 (84.8%) and 26/33 (78.8%) in all three epidemic waves, respectively. A mutation that had never been reported elsewhere was not considered a mutation of concern and was thus excluded for further analysis.

### 3.4. Clinical Characteristics of COVID-19 Patients in Three Epidemic Waves

The demographic and clinical information were obtained from 32 patients (data on one patient in the first wave remained unavailable) (Table 2). The median age of all patients was 37 years (range 1–74); 46.9% were male, and 53.1% were female. The distributions of age and gender in the first and second waves were similar; the median ages were 32 (13–48) and 34 (18–67) years, respectively. Females were affected more than males, whereas higher median age and number of males than females were observed in the third wave. The clinical features of patients in each wave were elaborated. In the first wave, the numbers of asymptomatic and symptomatic patients were comparable at 42.9% and 57.1%, respectively. The symptoms included mild upper respiratory indications (fever, cough, and sore throat) and diarrhea, which were relieved without hospitalization. The second wave caused more symptomatic indications compared to the first wave; 80% of patients presented symptoms, of which 60% had mild illnesses, and 20% developed severe-to-critical illnesses. One of two severe patients had underlying diseases, namely hypertension and diabetes. All of the patients in the second wave outbreak recovered. A notably more intense situation was observed in the third wave, in which the mass vaccination campaign had not yet started. Nearly all infected people had symptoms, particularly lower respiratory symptoms including cough, sputum production, and shortness of breath. All patients required hospital admission (isolation in a healthcare facility for an asymptomatic patient); 40% had moderate symptoms, and 33.3% developed severe-to-critical illnesses. Patients who had comorbidities (66.7%) such as hypertension, diabetes, and heart disease were affected more by this cluster and had a higher risk of death. The clinical outcomes of the third wave resulted in 11 (73.3%) recoveries and four (26.7%) deaths, all of which had underlying diseases.

### 3.5. SARS-CoV-2 Mutations Associated with Disease Severity

The mutations found in virus sequences retrieved from patients who had no symptoms, mild symptoms, and severe-to-critical illness were characterized and grouped by COVID-19 waves (Table 3). A majority of aa substitutions were lineage-defining mutations which were not likely associated with disease severity. For example, (i) S:A829T (A.6 lineage) was detected in asymptomatic and mild-symptom patients in the first wave; (ii) S:L5F, S459F, and D614G (B.1.36.16 lineage) were detected in asymptomatic and severe-to-critical patients in the second wave; (iii) ten aa substitutions in the S protein of B.1.1.7 were found in all patients with varying degrees of severity. However, some mutations were specifically identified in certain clinical spectra. For example, S protein T549I and envelope (E) protein S55F were detected only in severe-to-critical patients, who eventually died, suggesting that these mutations might be potentially pathogenic determinants of the virus which could have influenced disease severity.

## 4. Discussion

SARS-CoV-2 has placed a continuous strain on the global population with the emergence of several VOCs. Genomic surveillance has been implemented to track viral transmission, monitor mutations, evaluate the rate of evolution, and determine the potential for causing future outbreaks. From the beginning of the COVID-19 pandemic up to June 2021, Thailand experienced three COVID-19 waves that were dominated by distinct viral lineages. In the absence of herd immunity, the introduction of a new variant in each wave contributed to increases in the number of confirmed cases of COVID-19 as well as disease severity. The total case numbers and deaths in the third wave were nearly 30 times that from the first wave [10]. This fact led to the exploration of the genomic diversity of dominant viral lineages in the three waves using WGS. The correlation between mutations and clinical data was also analyzed. Whole-genome analyses revealed the differences in mutation patterns among predominant lineages, which might be associated with transmissibility, infectivity, and disease severity.

During the first wave, Pangolin lineages A.6, B, B.1, and B.1.375 were identified from the samples in the current study and linked to asymptomatic or mild symptoms. The results in this study were in line with those from the study of Puenpa J. et al., wherein clade L (Wuhan-Hu-1-like), S (A.6), G (B.1), V (B), and O (Others) were detected in samples collected from mild symptomatic patients during the first COVID-19 wave in Thailand [11]. Several research groups have attempted to link genetic variations with disease severity. The majority of mutations that occurred during the first wave have been shown to relate to asymptomatic and mild diseases. Nagy Á. et al. identified the mutations in viral sequences retrieved from the GISAID database and observed that NS8:L84S (A.6 lineage) and Nsp6:L37F (B lineage) had higher frequencies among asymptomatic and mild patients [19]. Similar to the study by Aiewsakul P. and colleagues, Nsp6:L37F was significantly associated with asymptomatic patients [20]. Moreover, structural analysis and ex vivo study of NS3:Q57H (B.1 lineage) provided evidence supporting that this mutation was associated with decreased viral virulence, resulting in decreased mortality rates and increased transmission [21,22].

The second wave of the COVID-19 surge was driven by the B.1.36.16 lineage. Most of the patients in the second wave had mild respiratory illnesses, which is consistent with an article from South China in which Xu H. and colleagues reported that patients infected with B.1.36.16 were more symptomatic than earlier strains [23]. High-frequency substitutions were S: D614G; N:S194L; Nsp6:L37F; Nsp12:P323L; NS3:Q57H, which were found in all samples collected during the second wave. Co-mutational combinations (S:D614G, NS3:Q57H, N:S194L) have been shown to correlate with mild and severe outcome based on a model predicting mutations-associated disease severity [24], indicating that the co-mutations were possibly responsible for symptomatic manifestations compared to the previous outbreak. The second wave of the COVID-19 surge was driven by the B.1.36.16 lineage. Most of the patients in the second wave had mild respiratory illnesses, which is consistent with an article from South China in which Xu H. and colleagues reported that patients infected with B.1.36.16 were more symptomatic than earlier strains [25,26]. For example, the combination of S:D614G and Nsp12:P323L has been proven to be an epidemiologically successful variant, giving an advantage to increase the viral fitness and transmissibility but not disease severity [27,28,29]. Our results showed that S:D614G and Nsp12:P323L were dominant in the second and third waves, respectively, suggesting that this combination might have significantly impacted enhanced transmission, contributing to increasing COVID-19 case numbers.

The predominant lineages of the third wave were designated as VOCs (Alpha variant; B.1.1.7 and its sub-lineages). This variant spread rapidly across the globe, increasing the risk of ICU admission and mortality, especially in among older people [30,31]. Data for the third wave showed the same trend as other countries in terms of clinical characteristics and the rise of daily COVID-19 confirmed cases [32,33,34]. The B.1.1.7 and Q3 lineages harbored mutation patterns that could facilitate transmissibility, infectivity, and immune evasion, particularly in the S protein such as del69-70HV, del144Y, N501Y, A570D, D614G, P681H, T761I, S982A, and D1118H. For example, N501Y increases the binding affinity to the host receptor; P681H enhances the spike cleavage, impacting the viral entry; del69-70HV is likely to be involved with immune evasion and an increase in viral transmission [35,36]. Mutations in non-spike proteins such as (N: D3L, R203K, G204R, S235F; Nsp3: T183I, A890D, I1412T; Nsp6: SGF106-108del; Nsp12: P323L; NS3: G254stop; NS8: Q27stop, R52I, K68stop, Y73C) were also observed at a high frequency (85–100%) corresponding to the global prevalence [37]. Although the potential effects of these mutated proteins have not been investigated thoroughly, the relationship among mutations may have provided transmissibility, infectivity, and replication advantages for the alpha variant, eventually making it predominant in Thailand. However, these signature mutations were not likely to contribute to enhanced disease severity since they were detected in patients with varying degrees of clinical symptoms and illnesses ranging from mild to critical.

In this study, a few additional mutations were found only in sequences retrieved from severe/critical and deceased patients. The aa substitutions in structural proteins might affect viral attachment and entry into host cells. The mutational effect of S:T549I has not been reported previously. However, position 549 on the S protein is proximal to RBD, which may affect the binding affinity to the ACE2 receptor and subsequently influence viral entry. S55F was predicted to improve the binding of the SARS-CoV-2 envelope protein to a tight junction-associated protein (PALS1) [38]. In addition, changes in the non-structural proteins might be involved with viral replication and the ability to counteract with host immune response [39]. For example, Nsp2 mutations might affect virulence by interfering with the interaction with host proteins [40]; Nsp3 mutations might be responsible for interacting with host immunity and contribute to unfavorable clinical outcome in patients [41]; and Nsp14 mutations possibly affect the genomic diversity and evolution of viruses [40]. Nevertheless, the functional impacts of these substitutions need to be investigated further. Due to the low frequency of occurrence, we postulated that these mutations probably change the virus phenotype to be more pathogenic and decrease viral fitness. It should be noted that the mutation-associated disease severity could vary widely due to the wide-ranging clinical information regarding symptom classification and patient management in each country.

Not only are genetic variations of the virus responsible for virulence, but the host factor also contributes to severe outcomes. The presence of pre-existing comorbidities including hypertension, heart disease, diabetes, and obesity combined with pathogenic determinants in the viral genome can cause detrimental effects on the host. Given the small sample size, determining the correlation between aa substitutions and clinical data is difficult. Further investigations including in vitro, in vivo, and structural analyses are required to explore whether additional mutations affect viral pathogenesis and disease severity since these mutations might rebound in subsequent emerging variants.

## 5. Conclusions

This study presented the genome characterization of SARS-CoV-2 variants that predominated during three COVID-19 waves in Thailand. The number of mutations increased over time and was directly proportional to the increase in confirmed cases as well as disease severity. The A.6, B, and B.1 lineages contained mutations that had minimal impacts on transmission; the lockdowns and preventive measures could have effectively controlled this wave. Accumulated mutations were observed in B.1.36.16, relative to the increase of symptomatic COVID-19 patients in the second wave. The alpha variant that was predominant in the third wave harbored mutations that were essential for enhanced transmissibility and infectivity. Thus, a sharp rise in case numbers and increased hospitalizations were recorded. All lineage-defining mutations were not likely to be associated with severe diseases since they were detected in sequences retrieved from patients in all clinical spectra. Nevertheless, six mutations were detected only in severe and deceased patients, suggesting that these additional mutations possibly gave rise to phenotypic changes to attain higher pathogenicity, especially in patients with underlying conditions. The findings of this study can provide more insights into the genomic epidemiology and diversity of SARS-CoV-2 circulating in Thailand.

## Figures and Tables

**Table 1:** Number of samples collected and lineage distribution at each wave.

Wave of Infections	n	Samples	Lineages (n)
First wave	8	VJR004, VJR007, VJR009, VJR010	A.6 (4)
		VJR008	B (1)
		VJR003, VJR005	B.1 (2)
		VJR002	B.1.375 (1)
Second wave	10	VJR022, VJR023, VJR024, VJR025, VJR026, VJR027, VJR028, VJR029, VJR030, VJR031	B.1.36.16 (10)
Third wave	15	VJR034, VJR037, VJR038, VJR039, VJR040, VJR041, VJR042, VJR043, VJR044, VJR045, VJR046, VJR047, VJR048, VJR050	B.1.1.7 (14)
		VJR035	Q.3 (1)
Total	33		

**Table 2:** Clinical characteristics of COVID-19 patients in three COVID-19 waves.

	Total Cases (n = 32)	First Wave (n = 7)	Second Wave (n = 10)	Third Wave (n = 15)
**Age (Years)**				
Median (range)	37 (1–74)	32 (13–48)	34 (18–67)	55 (1–74)
**Gender, n (%)**				
Male	15 (46.9)	3 (42.9)	2 (20)	10 (66.7)
Female	17 (53.1)	4 (57.1)	8 (80)	5 (33.3)
Symptoms, n (%) *				
Fever	15 (57.7)	2 (50)	4 (50)	9 (64.3)
Cough	16 (61.5)	2 (50)	3 (37.5)	11 (78.6)
Sore throat	5 (19.2)	2 (50)	2 (25)	1 (7.1)
Sputum production	8 (30.8)	-	2 (25)	6 (42.9)
Headache	3 (11.5)	1 (25)	1 (12.5)	1 (7.1)
Nasal congestion	4 (15.4)	-	2 (25)	2 (14.3)
Loss of taste and smell	2 (7.7)	-	1 (12.5)	1 (7.1)
Fatigue	4 (15.4)	-	1 (12.5)	3 (21.4)
Myalgia	5 (19.2)	-	2 (25)	3 (21.4)
Breathing difficulty	13 (50)	-	2 (25)	11 (78.6)
Diarrhea	3 (11.5)	1 (25)	-	2 (14.3)
Other	1 (3.8)	-	-	1 (7.1)
**COVID-19 disease severity, n (%)**				
Asymptomatic	6 (18.8)	3 (42.9)	2 (20)	1 (6.7)
Mild	13 (40.6)	4 (57.1)	6 (60)	3 (20)
Moderate	6 (18.8)	-	-	6 (40)
Severe to critical	7 (21.9)	-	2 (20)	5 (33.3)
**Hospitalization**				
No	13 (40.6)	7 (100)	6 (60)	0
Yes	19 (59.4)	0	4 (40)	15 (100)
**Comorbidities**				
Any *, n (%)	11 (34.4)	-	1 (10)	10 (66.7)
Diabetes	4 (12.5)	-	1 (10)	3 (20)
Hypertension	8 (25.0)	-	1 (10)	7 (46.7)
Obesity	1 (3.1)	-	-	1 (6.7)
Cancer	1 (3.1)	-	-	1 (6.7)
Cerebrovascular disease	2 (6.3)	-	-	2 (13.3)
Heart disease	3 (9.4)	-	-	3 (20)
**Final clinical outcomes, n (%)**				
Recovered	28 (87.5)	7 (100)	10 (100)	11 (73.3)
Deceased	4 (12.5)	-	-	4 (26.7)

* More than one symptom or comorbidity can be given.

**Table 3:** The association of amino acid substitutions with COVID-19 severity.

	First Wave		Second Wave			Third Wave			
	No Symptom	Mild	No Symptom	Mild	Severe/ Critical	No Symptom	Mild	Moderate	Severe/ Critical
**Structural Protein**									
**Spike**	A829T	A829T	L5F	S459F	L5F	H69del	H69del	V6F	H69del
		D614G	S459F	D614G	S459F	V70del	V70del	H69del	V70del
			D614G		D614G	Y144del	Y144del	V70del	Y144del
						N501Y	Y144F	Y144del	N501Y
						A570D	N501Y	N501Y	T549I
						D614G	A570D	A570D	A570D
						P681H	D614G	D614G	D614G
						T716I	P681H	P681H	P681H
						S892A	T716I	T716I	T716I
						D1118H	S892A	S892A	S892A
							D1118H	D1118H	D1118H
							Q784S		
							Q787H		
							I788V		
**Envelope**									S55F
**Nucleocapsid**	S187L		S194L	S194L	S194L	D3L	D3L	D3L	D3L
				D402Y		R203K	R203K	R203K	R203K
						G204R	G204R	G204R	G204R
						S235F	S235F	S235F	S235F
								247–251 del	
**Non-structural protein**									
**Nsp1**							L16V		
**Nsp2**		A476V	E373R						G47S
**Nsp3**	L557F	L557F	D164G	D164G	P1200S	T183I	T183I	T183I	T183I
		T583I	P1200S	P1200S		A890D	A890D	A890D	A890D
			T860del	L1195I		I1412T	I1412T	I1412T	I1412T
			A861R	V393F		M1529I		Q1530H	D1755N
			L862M	T943N		Q1530E		A894del	
			F1110I			F1532V		L895V	
**Nsp6**	L37F	L37F	L37F	L37F	L37F	S106del	S106del	S106del	S106del
				Q160K		G107del	G107del	G107del	G107del
						F108del	F108del	F108del	F108del
									A54del
**Nsp9**				T109I					
**Nsp12**		P323L	P323L	P323L	P323L	P323L	P323L	P323L	P323L
**Nsp13**			T588I	T588I	T588I			W167M	W167M
				A18V					
				V232I					
**Nsp14**			T215S	T215S	W227R			S369F	
			T215R	217–226 del					
			216–227 del						
**Nsp15**	V172L	V172L		D282N					
**Nsp16**				D114del			D293G		
							V294I		
**NS3**		G251V	Q57H	Q57H	Q57H	G254stop	G254stop	G254stop	G254stop
		Q57H		G254stop			I232L		
		S216P		V55F					
		V13L		D27Y					
				I232L					
**NS7b**	C41F								
**NS8**	L84S	L84S				Q27stop	Q27stop	Q27stop	Q27stop
		D75Y				R52I	R52I	R52I	R52I
						K68stop	K68stop	K68stop	K68stop
						Y73C	Y73C	Y73C	Y73C

## Data Availability

The data presented in this study are not publicly available due to confidentiality but are available on reasonable request.

## Supplementary Materials

The following supporting information can be downloaded at: https://www.mdpi.com/article/10.3390/pathogens12040626/s1, Table S1: Global prevalence of amino acid substitutions identified in our study.

## Abbreviations

1st	first
2nd	second
3rd	third
aa	amino acid
ACE2	angiotensin-converting enzyme 2
cDNA	complementary DNA
COVID-19	coronavirus disease 2019
del	deletion
DNA	Deoxyribonucleic acid
GISAID	Global Initiative on Sharing Avian Influenza Data
NPS	nasopharyngeal swab
Nsp	nonstructural protein
RBD	receptor-binding domain
RNA	Ribonucleic acid
S protein	spike protein
SARS-CoV-2	severe acute respiratory syndrome coronavirus 2
ssCirDNA	circularized single strand DNA
VOC	variants of concern
VOI	variants of interest
WGS	whole-genome sequencing
