# Supplementary material for: SARS-CoV-2 Variants by Whole-Genome Sequencing in a University Hospital in Bangkok: First to Third COVID-19 Waves
Source: Pathogens. 2023 Apr 21;12(4):626. doi: 10.3390/pathogens12040626 (PMC10146024; doi:10.3390/pathogens12040626)
Supplement: Supplementary file 1 [file pathogens-12-00626-s001.zip › pathogens-2260654-supplementary.pdf]

Supplementary data

Supplementary Table S1: Global prevalence of amino acid substitutions identified in our study

Protein	Mutation	No. of Occurrence (Global)	No. of Countries with This Mutation	Global Frequency * (%)
Structural protein				
**Spike**	A829T	974	34	0.0174
	D614G	5432315	204	97.2442
	L5F	140590	161	2.5167
	S459F	2181	40	0.0390
	V6F	4695	62	0.0840
	H69del	1138065	179	20.3726
	V70del	1139366	179	20.3959
	Y144F	2173	62	0.0389
	Y144/145del	1125931	181	20.1554
	N501Y	1296558	189	23.2098
	T549I	1075	28	0.0192
	A570D	1144986	181	20.4965
	P681H	1221392	181	21.8642
	T716I	1144025	176	20.4793
	Q784S	80	8	0.0014
	Q787H	110	19	0.0020
	I788V	188	16	0.0034
	S982A	1112492	171	19.9148
	D1118H	1113427	172	19.9315
**Envelope**	S55F	3757	70	0.0673
**Nucleocapsid**	S187L	14932	80	0.2673
	S194L	97908	141	1.7527
	D402Y	7933	92	0.1420
	D3L	1104849	179	19.7780
	R203K	1491718	195	26.7033
	G204R	1408936	195	25.2214
	S235F	1116443	178	19.9855

	T247del	41	13	0.0007
	K248del	58	17	0.0010
	K249del	56	17	0.0010
	S250del	58	18	0.0010
	A251del	66	18	0.0012
**<u>Non-structural protein</u>**				
**Nsp1**	L16V	9	3	0.0002
**Nsp2**	A476V	6429	78	0.1151
	E373R	1	1	0.0000
	G47S	696	36	
**Nsp3**	L557F	946	30	0.0169
	T583I	1348	50	0.0241
	D164G	907	38	0.0162
	P1200S	6349	78	0.1137
	T860del	30	8	0.0005
	A861R	1	1	0.0000
	L862M	9	4	0.0002
	F1110I	116	22	0.0021
	L1195I	74	8	0.0013
	V393F	2442	50	0.0437
	T943N	118	11	0.0021
	T183I	1115497	180	19.9686
	A890D	1116403	176	19.9848
	I1412T	1091091	175	19.5317
	Q1530H	21	12	0.0004
	A894del	16	6	0.0003
	L895V	2	2	0.0000
	D1755N	122	18	0.0022
	Y1082L	29	4	0.0005
	K1083R	319	19	0.0057
	M1529I	2191	49	0.0392
	Q1530E	6	9	0.0001
	F1532V	26	9	0.0005

**Nsp6**	L37F	137239	164	2.4567
	Q160K	3700	45	0.0662
	S106del	1331115	187	23.8284
	G107del	1331224	187	23.8303
	F108del	1328374	187	23.7793
	A54del	4699	55	0.0841
**Nsp9**	T109I	8816	88	0.1578
**Nsp12**	P323L	5333320	203	95.4721
**Nsp13**	T588I	11541	105	0.2066
	A18V	7166	89	0.1283
	V232I	409	22	0.0073
	W167M	2	2	0.0000
**Nsp14**	T215S	13	4	0.0002
	T215R	6	3	0.0001
	W227R	7	4	0.0001
	C216del	48	12	0.0009
	F217del	51	12	0.0009
	S218del	68	13	0.0012
	T219del	72	16	0.0013
	A220del	75	16	0.0013
	S221del	75	18	0.0013
	D222del	77	20	0.0014
	T223del	71	18	0.0013
	Y224del	58	15	0.0010
	A225del	56	16	0.0010
	C226del	48	12	0.0009
	W227del	47	14	0.0008
	S369F	1791	57	0.0321
**Nsp15**	V172L	4438	337	0.0794
	D282N	610	37	0.0109
**Nsp16**	D114del	22	6	0.0004
	D293G	0	0	0.0000
	V294I	884	37	0.0158

**NS3**	G251V	7769	77	0.1391
	Q57H	497302	190	8.9022
	S216P	1271	51	0.0228
	V13L	8318	94	0.1489
	G254stop	7379	78	0.1321
	I232L	25	8	0.0004
	V55F	5606	85	0.1004
	D27Y	27950	3	0.5003
**NS7b**	C41F	4042	77	0.0724
**NS8**	L84S	16155	674	0.2892
	D75Y	529	32	0.0095
	D75N	697	38	0.0125
	Q27stop	1118248	178	20.0178
	R52I	1116858	181	19.9929
	K68stop	384613	159	6.8850
	Y73C	1118494	177	20.0222

* Global frequency is retrieved on December 6, 2021
